# Nurses’ attitudes towards enforced measures to increase influenza vaccination: A qualitative study

**DOI:** 10.1111/irv.12441

**Published:** 2017-01-31

**Authors:** Anina Pless, David Shaw, Stuart McLennan, Bernice S. Elger

**Affiliations:** ^1^ Institute for Biomedical EthicsUniversity of BaselBaselSwitzerland; ^2^ Institute for History, Ethics and Philosophy of MedicineMedizinische Hochschule HannoverHannoverGermany; ^3^ Center for Legal MedicineUniversity of GenevaGenevaSwitzerland

**Keywords:** enforced measures, healthcare workers, influenza, nosocomial infection, qualitative research, vaccination

## Abstract

**Background:**

Despite studies demonstrating that the annual influenza vaccination of healthcare workers reduces morbidity and mortality among vulnerable patients, vaccination rates remain very low, particularly in nursing staff. Educational programmes have failed to improve rates, which has led to a diverse range of enforced approaches being advocated and implemented.

**Objectives:**

To examine the attitudes of non‐vaccinated nursing staff towards various enforced measures aimed at increasing rates of influenza vaccination.

**Methods:**

Semi‐structured qualitative interviews with a purposive sample of 18 non‐vaccinated nurses, working in units with high‐risk patients at two hospitals in Switzerland. Analysis of interviews was done using conventional content analysis.

**Results:**

Nurses were critical of enforced measures. However, measures that include an element of choice were perceived as more acceptable. Declination forms and mandatory vaccinations as part of the employment requirements were found to be the most accepted measures.

**Conclusion:**

The perception of choice is crucial to the acceptance of a measure. Respect for choice and autonomy has a positive effect on behavioural change.

Mandatory influenza vaccination as a condition of new (and perhaps ongoing) employment could be a feasible, effective and ethical measure to increase vaccination rates among nurses who oppose vaccination.

## 1 Introduction

Despite long‐standing recommendations by public health authorities and studies stating that annual influenza vaccination of healthcare workers (HCWs) is associated with a reduction of morbidity and mortality among patients,^1^ influenza vaccination rates among HCWs remain low internationally.^2^, ^3^, ^4^ Vaccination rates among nurses are typically lower than those of physicians.^5^, ^6^ While studies show interventions focusing on providing information and easier access to the vaccine have increased vaccination rates among physicians, they seem to have very little effect on nursing staff.^7^, ^8^ This has led a number of hospitals to implement more authoritarian approaches, including declination forms, mandatory wearing of masks, unpaid leave during influenza season, switching to another lower‐risk unit, and mandatory vaccination for nurses declining influenza vaccination.^9^, ^10^, ^11^, ^12^, ^13^, ^14^, ^15^, ^16^ Declination forms and mandatory masks for non‐vaccinated HCWs alone have resulted in minimal increases of vaccination rates.^13^, ^14^ Mandatory vaccination of HCWs has been shown to be the most effective measure, achieving almost universal coverage and very low refusal rates.^17^, ^18^ While mandatory vaccination raises issues concerning HCWs autonomy, it is increasingly considered to be ethically justifiable.^15^, ^19^, ^20^, ^21^ Interestingly, the attitudes of HCWs towards mandated vaccinations are not as critical as might be assumed.^20^, ^22^, ^23^ Several studies in the United States and Europe show that a majority of HCWs agree that influenza vaccination for HCWs should be mandatory and that they would accept mandatory measures under certain circumstances.^24^, ^25^, ^26^, ^27^ Questions remain concerning how the implementation of measures aimed at improving vaccination rates would be accepted, particularly among nurses.

The aim of this study was to explore the attitudes of non‐vaccinated nursing staff, working in units with patients at high risk of morbidity and mortality of influenza towards various enforced measures aimed at increasing rates of influenza vaccination. We chose nurses working in units with high‐risk patients because we assumed these nurses would be more aware of the danger they would possibly present to their patients by refusing the influenza vaccination. We hope to identify common reactions of nursing staff towards enforced measures to improve influenza vaccination by letting them discuss the issue. Better understanding of their attitudes could help to guide interventions and policy recommendations aimed at increasing vaccination rates.

## 2 Methods

The study was approved by the Ethics Committee of the Basel Cantons on the 27th of January 2012. All participants gave oral informed consent.

### 2.1 Setting and recruitment procedures

Non‐vaccinated participants were recruited from several nursing departments in two teaching hospitals in the German‐speaking part of Switzerland. The administrators of the different departments were contacted in February 2012 by e‐mail. Those willing to participate were asked to name possible interviewees. Additional participants were acquired using a snowball approach, particularly through well‐connected interviewees. Purposive sampling was employed to ensure that nurses were from a range of fields, hierarchical positions and work experience. Participation was entirely voluntary. Interviewees were granted full confidentiality and anonymisation of any personal identifiers or situations in interview quotes.

### 2.2 Data collection

Interviews were conducted during spring and fall 2012. In order to minimise bias, we let the participants choose the setting of the interviews: most chose their workplace, but some interviews took place in public places. Only A.P. and the respective interviewee were present at the interviews. There was no relationship between the investigator and the participants prior to the study and the participants knew only that the investigator was a student of medicine and that the topic of the study was mandatory measures to increase influenza vaccination. They had no knowledge of the investigator's position on the topic and none of the interviewees asked before or during the interview. Three interview partners asked about the investigator's views on mandatory influenza vaccination after the interview. Interviews lasted an average of 30 minutes and were audio‐recorded. In addition, field notes were made by the investigator shortly after the interviews. These included notes on how the interviews may have been influenced by the investigator, that is by follow‐up questions asked or by verbal or non‐verbal reactions to what the interview partners had said. Interviews were conducted in Swiss German or High German, depending on the participant's preference. All recordings were transcribed verbatim using High German diction, as there is no standard diction of Swiss German and we strove to make transcripts consistent. A.P., who conducted and transcribed the interviews and is a native Swiss German speaker, also translated the Swiss German interviews. Analysis was conducted using the High German transcription. Language barriers between researchers and participants can pose a methodological challenge. Our approach largely met Squires' recommendations for cross‐language qualitative research.^28^

Demographic details were gathered prior to the interviews. A semi‐structured interview guide regarding nurses' attitudes about enforced measures to increase influenza vaccination was created to give a frame to the conversation and follow‐up questions were asked based on the interviewees' responses. It was tested in the first interview, after which a follow‐up discussion among the research team deemed it applicable. No repeat interviews were carried out and the transcripts were not returned to participants.

### 2.3 Data analysis

A.P., the investigator who conducted the interviews and did the primary analysis, was a master's student of medicine as well as a student of cultural anthropology and history at the time of the study. She thus had knowledge in the field of medicine, as well as basic theoretical and practical knowledge of qualitative research. Conventional content analysis was performed by the investigator who conducted the interviews [A.P.], whereby data were read and reread for emergent themes and relationships and any themes, categories or properties that appeared in the data were compared to earlier data as well as to the research literature on the subject.^29^ Initial themes discovered in the transcribed interviews were labelled using a process of open coding in order to identify, describe or categorise phenomena in the data.^29^, ^30^, ^31^ The investigator A.P. conducted the open coding and analysis together with S.M., B.E. and D.S., who all have long‐standing training and experience in the field of qualitative research. During and following open coding, higher‐order codes were identified that further elucidated important meanings and explanations. Coding differences were resolved to achieve consensus and the investigators agreed that saturation was reached after 18 interviews and that all the important themes and views had been touched upon in the interviews. The research team discussed their own positions on the topic and the field notes throughout the research process. The interviewed nurses knew that the investigator was a medical student and this possibly had an effect on their answers during the interview. The research team found no clear indicators of this in the interviews or the field notes but agreed that while some nurses possibly were more cautious in expressing their views due to this knowledge, on others this may have had the opposite effect of speaking more frankly. Also, the members of the research team generally believed in the importance of influenza vaccination in the healthcare setting and some members were supportive of mandatory measures to increase influenza vaccination. The possible impact of the research teams' prior assumptions and beliefs on the study findings was discussed throughout the research process and we critically re‐examined our study findings with this in mind until consensus was reached that the influence was only minimal. None of the interviewees dropped out of the study. After completion, all participants were provided with an electronic version of the results and discussion.

## 3 Results

### 3.1 Participants/nurses characteristics

A total of 18 nurses were interviewed, 14 were female. Participants' work experience ranged from 1 to 37 years (mean 14.4, median 7.5). Nurses worked in six different units with patients at high risk of morbidity and mortality due to influenza (haematology, cardiology, nephrology, geriatrics, ICU, oncology) and held various hierarchical positions. Of the eight department heads contacted via e‐mail, seven replied. One department head declined to participate, six agreed to ask their staff to take part and contact the research team. Approximately 25 nurses contacted the researchers, of which 2 declined to take part before a meeting was set. Of the two nurses who declined to take part, one named lack of time as the reason, and the other did not give a reason for deciding not to take part. Seventeen nurses spent more than half of their time working with patients directly. None of the nurses had previous experience with the enforced measures to increase influenza vaccination, which were explored in the interviews.

### 3.2 Attitudes towards enforced measures

Nurses' attitudes to enforced measures varied depending on their general position regarding vaccination or influenza vaccination specifically and also depending on the particular enforced measure in question,

#### 3.2.1 Mandatory mask wearing

The idea of compulsory mask wearing during the influenza season for nurses who refuse to get the influenza vaccine was universally criticised by participants. Many felt this measure to be stigmatising and discriminating and they believed such a measure would cause tension in the team because it would make vaccination status visible and thus create a divide among coworkers. Also, they thought it would alienate and irritate patients, who might not understand why some nurses wore a mask and others did not and might also refuse to be attended to by nurses who wore masks, marking them as “bad nurses.”

This… this is a bit far fetched and exaggerated, but it reminds me of the Yellow star… Stigmatising. That was a little crass, but that was the first thing that came to my mind. (HCW 8)

Nonetheless, the majority of nurses answered that if this were to be implemented in their institution, they would wear the mask rather than be inclined to get the vaccination. This was often deemed as choosing “the lesser of two evils.” Some nurses who were ambivalent about getting the vaccine stated that being confronted with this enforced measure fortified their reluctance to get vaccinated.

Well, I think I would just have to adapt. But it wouldn't be a reason for me to get vaccinated. (HCW 18)

For those nurses accustomed to wearing masks during their daily line of work (i.e isolation ward), wearing a mask at work was not a major issue. They argued that they wear masks anyway. However, several other nurses feared the discomfort this measure would entail. For some, mandatory mask wearing would prompt them to get the influenza vaccination.

Well, then I would think about it again I guess, whether to get vaccinated. Because we all know what it's like to go into the isolation unit with the masks and all that and, even if it's just for fifteen minutes it's… and then you have to walk around with the mask all day. (HCW 10)

#### 3.2.2 Declination forms

When asked about declination forms, reactions were divided; some nurses said they would sign the declination form and not get the vaccination, and more often interviewees said they would let themselves be vaccinated if this measure were to be implemented. Generally, however, this measure was regarded as acceptable, since it still left it up to the employees to decide whether they got the vaccination or signed a declination form. Some interviewees also thought this to be a good approach since it encouraged people to think about their reasons and thus make a more educated decision, which was seen as an advantage over mandatory mask wearing.

I think that would be the best solution, this opt‐out. People would definitely think about it more. Because they would have to fill that in, they would have to elaborate their reasons. And then they would maybe realise: “I don't have any reasons.” (HCW 16)

Furthermore, a couple of participants stated that use of declination forms would raise the vaccination rate in general, since those people who are unsure whether to get vaccinated, too lazy to do so or simply have not given the issue much thought would be more inclined to comply if they were confronted with the inconvenience of having to sign a form.

Yes, (…) some people who just take the easier road or are just a little minimalistic would think: “Okay, then I'll just get the vaccination quickly. I mean it's just a matter of a couple of seconds. Then I don't have to deal with these papers and stuff.” When I think about it, the majority of people in our unit would say that, yes. (HCW 9)

On the other hand, participants mentioned the fear of consequences of signing such a declination form: if a patient were to be infected subsequently, they might be held accountable for the transmission of the virus.

#### 3.2.3 Switching units

Many of the participants believed a mandate for non‐vaccinated nurses to switch to units with less vulnerable patients would lead to problems with staff shortage since many units with high‐risk patients depend on specialised nursing staff.

A big question mark. Especially the isolation unit and of course highly specialised units, which require trained personnel. They would be in distress, real distress. (HCW 2)

However, only a few nurses said they would consequently switch to another unit or thought others would do so. One nurse stated this would be a reason for her to quit the profession. Several nurses clearly said if this measure were to be implemented, they would get the vaccination; their work was too important to them and this measure was mostly deemed as acceptable, since it still left it up to nurses whether they got vaccinated or switched to another unit.

I would think about it, whether to get the vaccination instead of switching to another unit. (…) I also probably would say: “Okay, then I'll just get the vaccination.” (HCW 10)

Others agreed it would be reasonable to get vaccinated when working with certain high‐risk patients; however, most often they did not perceive their own patients as belonging to this vulnerable group. Although only nurses working with high‐risk patients were interviewed, most nurses did not see their rejection of the influenza vaccination as posing a threat for their own patients.

And I have to say, if I were working with leukaemia patients or something, I obviously would get vaccinated. There, the risk is evident. (HCW 9)

#### 3.2.4 Mandatory vaccination/condition of employment

Although all nurses emphasised that it went against their conviction, the majority of nurses said they would get the vaccination if mandatory vaccination were to be implemented in their institution. For most, this was the better alternative to losing their job or pursuing a new career. They were particularly likely to submit to this measure if they were content with their workplace and its conditions otherwise.

If everything else is right, I don't think it would be a reason to quit. No, I don't think so. It would just be that way. (HCW 10)

Few nurses said they would quit or get fired and some warned that implementing measures like this would be bad for the reputation of their profession and would discourage young people from choosing this path. Half of the participants believed those implementing mandatory vaccination would encounter legal obstacles. They expected demonstrations or involvement of unions or lawyers.

That would definitely raise the rate, but I don't think that would be feasible. Because of the opposition of the unions and who knows who else… they would come. Whether it's even feasible by law or human rights or whatever. (HCW 6)

On the other hand, some—albeit fewer—interviewees doubted there would be much opposition. Although one might expect people to revolt and some might indicate this beforehand, they thought most people would just comply in the end.

I wouldn't go demonstrating either. (…) We didn't even go demonstrating for better wages or something like that. People would just get vaccinated. I think if they were to take drastic measures then you could somehow impose that. Because I'm thinking about it and nurses, (…) I don't really have the feeling that we're that fierce. We're more harmonious. Yes, then I would also just hold out my arm and… yes. (HCW 17)

Another theme, which was often mentioned, was influenza vaccination as an initial condition of employment. Most interviewees found this measure to be acceptable. It would still leave the decision to the employee, since the contracts would clearly state this condition from the beginning. This way, mandatory influenza vaccination would simply become part of the job description. No nurses said having mandatory influenza vaccination in their contract from the beginning would have influenced their choice of profession or hindered them from applying for their job.

I find that great. Because it's not the only vaccine you have to get. I have to get an HIV test done, that's a condition. I have to get vaccinated against rubella, that's a condition. Why not the flu shot? (HCW 14)

## 4 Discussion

To our knowledge, we have conducted the first qualitative study in Europe of nurses' attitudes towards enforced measures to increase influenza vaccination. Our results are particularly important as it provides a better understanding of attitudes in regions where vaccination rates are traditionally low and mandatory measures are looked upon critically.^32^ Our study found that the perception of choice was crucial to the acceptance of enforced measures. The interviewed nurses consider mandatory influenza vaccination as a condition of employment acceptable and perceive it feasible, effective and ethical.

While nurses who had previously declined influenza vaccination did not support the introduction of enforced measures—indeed, German‐speaking Switzerland is known for relatively high rates of general opposition of vaccination, including influenza vaccination^32^—our interviews showed that enforced measures are more widely accepted than might be expected.^13^, ^20^, ^33^ Although reluctant to comply, most nurses are not willing to give up their profession or work in a particular hospital only because of general opposition to vaccinations. Interestingly, the protection of patients was not mentioned or played only a minor role in the narratives and personal justifications provided by our participants. This has been reported in studies from other countries as well: patient protection does not seem to be a priority for nurses when confronted with the issue of influenza vaccination.^34^, ^35^ Moreover, there was no discourse on competing ethical values among our participants.

Our finding that the perception of choice was crucial to the acceptance of a measure warrants further analysis. Mandatory mask wearing for unvaccinated nurses and imposition of a mandatory vaccination policy were perceived much less acceptable than declination forms, the option to switch units and mandatory vaccination as a condition of employment. Hospitals are well advised to take into account these findings.

Almost all study participants perceived mandatory wearing of masks for non‐vaccinated healthcare workers as a form of unfair discrimination and even harassment. It became apparent that for the participants, restricting choices of non‐vaccinated HCW were not proportionate responses to protect patient interests, but rather unfair discrimination. Experiences at the University Hospital of Geneva and the University Hospital of Frankfurt have shown that the mandatory wearing of masks correlates with an increase in vaccination rates.^13^ The weakness of this measure is that there is no strong evidence that masks prevent influenza transmission.^36^, ^37^ One could argue that the main benefit is indirect, in that the inconvenience to wear a mask increases the acceptance of vaccination.

Influenza vaccination as a requirement of employment was much less criticised compared to mandatory vaccination. Nurses who were interviewed were already employed and may have had the perception that this measure would therefore not be applicable to them. However, our study showed that this interpretation is too simple: mandatory vaccination as a condition of employment was considered an acceptable option mainly because participants saw it as leaving them with more freedom of choice. This finding is interesting because in practice the two measures would have the same effect: in the end everybody working in an institution would have to submit to the vaccination.

The fact that the two measures were nonetheless perceived very differently indicates that a large part of HCW vaccination resistance also stems from the way measures are implemented and how the implementation is proposed. None of the nurses interviewed said they would have chosen a different profession if influenza vaccination had been required for employment. In a working environment where many work‐related tasks are dictated to them, a certain amount of autonomy seems essential to nurses. Many of the interviewed nurses thought that too much was being asked from them in general, they were unwilling to “give more,” particularly as they did not receive much recognition in return. Moralising pressure by authorities, especially enforced measures to increase influenza vaccination, seems to lead to more emphasis on autonomy and thus rejection of vaccination. Previous studies have pointed out the importance of recognition and autonomy when one tries to obtain change in vaccination‐related attitudes and behaviour^27^, ^33^ The results from our study suggest that measures, which leave nurses with some decisional autonomy, are more acceptable than measures which are merely decreed. While it may be helpful to convince nurses to attribute a higher priority to patients' health, this “moralising” approach might be insufficient. It is important to take into account nurses' perception that their autonomy is not respected and address it when planning future interventions. Therefore, nursing professionals' self‐empowerment as well as nurses' evidence‐based decision‐making skills should be promoted.

The question remains how making vaccination a condition of employment would work in practice, in particular for already employed workers rejecting the vaccine? This problem needs to be addressed before implementing such a measure.

The aim of this study was to gain insight into the attitudes of hesitant nurses towards such measures, thus obtaining a better understanding of barriers to and consequences of enforced measures in order to design new and more efficient interventions to increase vaccination in HCWs, who often have the closest contact to patients.

In summary, we found that the perception of choice is crucial to the acceptance of a measure. Respect for choice and autonomy has a positive effect on behavioural change.

The filling in of declination forms or mandatory vaccinations as a condition of employment seemed to be the most accepted enforced measures. Since declination forms have been shown to be of less effect on overall patient protection,^38^ mandatory influenza vaccination as a condition of new (and perhaps ongoing) employment could be a feasible, effective and ethical measure to increase vaccination rates among nurses who oppose vaccination.

## 5 Limitations

Like all interview studies, this research relied on consenting participants, increasing the chance of a biased sample; nurses who came forward may have been more likely to be unvaccinated nurses with a more pronounced opinion on this topic. In addition, thoughts on likely reactions to enforced measures were hypothetical, and it could be argued that their validity is therefore limited. However, the findings illustrate attitudes of nurses towards enforced measures and may shed light on actual reactions if new policies are introduced. It can be assumed that reactions would tend to be less pronounced in reality than in theory, as actually quitting a job or a profession with all the consequences this entails is most probably more difficult than saying one would do so. The small sample may limit the generalisability of our findings, but unlike in quantitative research, an adequate sample in qualitative research is not defined by the number of participants but relies on data saturation, meaning that all the important topics have been touched upon in the data collected. In our study, the researchers reviewed the material and agreed that after 18 interviews all the important themes and views had been touched upon and that further interviews would not bring more information.

## Conflict of Interest

All authors declare no actual or potential conflict of interest including any financial, personal or other relationships with other people or organisations within 3 years of beginning the work submitted that could inappropriately influence or bias this work. The study was funded internally within the University of Basel.
